# Antifungal Resistance and New Strategies to Control Fungal Infections

**DOI:** 10.1155/2012/713687

**Published:** 2011-12-01

**Authors:** Patrick Vandeputte, Selene Ferrari, Alix T. Coste

**Affiliations:** Institute of Microbiology, University of Lausanne and University Hospital, Rue du Bugnon 48, 1011 Lausanne, Switzerland

## Abstract

Despite improvement of antifungal therapies over the last 30 years, the phenomenon of antifungal resistance is still of major concern in clinical practice. In the last 10 years the molecular mechanisms underlying this phenomenon were extensively unraveled. In this paper, after a brief overview of currently available antifungals, molecular mechanisms of antifungal resistance will be detailed. It appears that major mechanisms of resistance are essential due to the deregulation of antifungal resistance effector genes. This deregulation is a consequence of point mutations occurring in transcriptional regulators of these effector genes. Resistance can also follow the emergence of point mutations directly in the genes coding antifungal targets. In addition we further describe new strategies currently undertaken to discover alternative therapy targets and antifungals. Identification of new antifungals is essentially achieved by the screening of natural or synthetic chemical compound collections. Discovery of new putative antifungal targets is performed through genome-wide approaches for a better understanding of the human pathogenic fungi biology.

## 1. Introduction

The fungal kingdom encompasses an enormous diversity of taxa with varied ecological niches, life-cycle strategies, and morphologies. However, little is known of the true biodiversity of Kingdom Fungi. Of the 1.5 million species estimated to belong to this kingdom, only about 5% were formally classified. Many fungi are parasites for plants, animals, human, and other fungi. Plant pathogenic fungi are able to cause extensive damage and losses to agriculture and forestry including the rice blast fungus, Dutch elm disease, and chestnut blight. Some other fungi can cause serious diseases in humans, several of which may be fatal if left untreated. These include aspergillosis, candidosis, coccidioidomycosis, cryptococcosis, histoplasmosis, mycetomas, mucormycosis, and paracoccidioidomycosis. The so-called dermatophytic and keratinophilic fungi can attack eyes, nails, hair, and especially skin and cause local infections such as ringworm and athlete's foot. Fungal spores are also a cause of allergies, and fungi from different taxonomic groups can provoke allergic reactions. In this paper, after a brief presentation of the medical impact of fungal infections at the global level and a summary of clinical treatments available today for clinicians, we will review the mechanisms underlying *in vitro* resistance to antifungals in fungal species of major importance in human medicine. Lastly, an overview of ongoing research undertaken to develop new therapeutic strategies to fight against fungal infections will be exposed.

## 2. Fungal Infections, Clinical Treatments, and Incidence of Antifungal Drug Resistance

### 2.1. Fungal Infections

At the beginning of the 20th century, bacterial epidemics were a global and important cause of mortality. In contrast, fungal infections were almost not taken into account. Since the late 1960s when antibiotic therapies were developed, a drastic rise in fungal infections was observed, and they currently represent a global health threat. This increasing incidence of infection is influenced by the growing number of immunodeficient cases related to AIDS, cancer, old age, diabetes, cystic fibrosis, and organ transplants and other invasive surgical procedures.

These infections are caused by two types of microorganisms: primary and opportunistic pathogens. Primary pathogens are naturally able to establish an infection in the healthy population. In contrast, opportunistic pathogens, among them commensal microorganisms of the healthy population, are able to develop infectious colonization of the human body when particular criteria, such as immunosuppression, are met. Fungal pathogens can be divided into two major groups: filamentous fungi and yeasts. Most of the primary pathogens are filamentous fungi, while most of the opportunistic pathogens are yeasts and some species of filamentous fungi are increasingly identified as opportunistic pathogens. It is also important to note that fungal infections can be classified in function of the tissue infected (see Table 1).

Superficial mycoses, such as* tinea versicolor*, are limited to the most external part of the skin and hair. These infections are most frequently caused by the species *Malassezia globosa *and* M. furfur*, which are estimated to be carried by 2% to 8% of the healthy population worldwide but could lead to *tinea versicolor* in some conditions that are still unclear [1].

Cutaneous and subcutaneous mycoses caused by dermatophytes fungi affect keratinized structures of the body. The most frequently involved dermatophyte genera are *Trichophyton*, *Epidermophyton*, and *Microsporum*. In most cases, cutaneous fungal infections require a challenge of immune system, and their incidence varies depending on the site of infection. For example, onychomycoses are very frequent in the global population, with an incidence varying from 5 to 25% [2].

Mucosal infections are mostly caused by opportunistic yeasts, and those belonging to the *Candida* genus are by far the most frequent. Vaginal, esophageal, oropharyngal, and urinary tract candidiasis are very frequent in immunocompromised patients. For example, esophageal candidiasis is associated with the entry into the clinical phase of AIDS and during the 1980s more than 80% of seropositive patients developed such an infection [3]. Fungal infections, of the eye are also classified as mucosal fungal infections, but are caused more frequently by *Fusarium* or *Aspergillus* species rather than *Candida* species.

Theoretically systemic mycoses may involve any part of the body, and a lot of species formerly considered as nonpathogenic are now recognized opportunistic pathogens responsible for deep-seated mycoses. These infections, with symptoms ranging from a simple fever to a severe and rapid septic shock, are very common in immunocompromised patients and are frequently associated with an elevated mortality rate. The most frequent pathogens involved in systemic fungal infections are *Candida*, *Pneumocystis*, *Histoplasma*, *Aspergillus*, *Cryptococcus, Mucor*, *Rhizopus*, and *Coccidioidomyces *[4–6].

### 2.2. Antifungal Agents

Despite extensive research dedicated to the development of new therapeutic strategies, there are only a limited number of available drugs to fight against invasive fungal infections. Indeed, only four molecular classes that target three distinct fungal metabolic pathways are currently used in clinical practice to treat essentially systemic fungal infections: fluoropyrimidine analogs, polyenes, azoles, and echinocandins. Several other classes, such as morpholines and allylamines are only used as topical agents due to either poor efficacy, or severe adverse effects when administered systemically.

#### 2.2.1. Fluoropyrimidines

**Figure 1:**
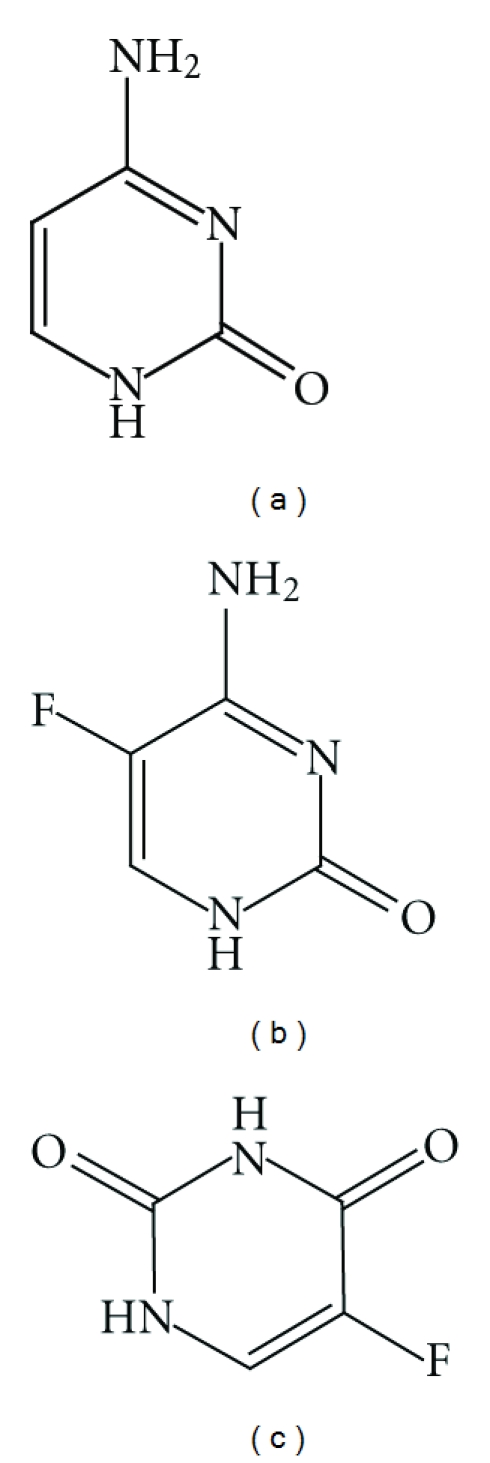
Chemical structures of cytosine (a) and of two fluoropyrimidines, 5-fluorocytosine (b), and 5-fluorouracil (c).

Fluoropyrimidines, of which only 5-fluorocytosine (5-FC) and 5-fluorouracil (5-FU) are used in human medicine, are synthetic structural analogs of the DNA nucleotide cytosine (Figure 1).

5-FC was synthesized in 1957 by Duschinsky et al., initially as an antitumor therapy [7]. In 1963, Grunberg and coworkers discovered its antifungal potential by means of murine models of cryptococcosis and candidiasis [8]. Several years later 5-FC was successfully used for the treatment of systemic candidiasis and of cryptococcal meningitis [9].

5-FC possesses a broad range of activity. This drug is active against *Candida* and *Cryptococcus* genera. 5FC activity on *Phialophora*, *Cladosporium,* and *Aspergillus* genera is much less limited. 5-FC is also active against protozoa belonging to *Leishmania* and *Acanthamoeba* genus [10].

Due to its high hydrosolubility and small size, 5-FC possesses interesting pharmacokinetic properties, since it diffuses rapidly throughout body even when orally administered [12]. Generally, it produces negligible side effects, despite some severe adverse effects, such as hepatotoxicity or bone marrow lesions [11, 13–15], occurring in rare cases [16]. Surprisingly, these side effects are identical to those observed with 5-FU treatment, despite the fact that humans do not possess a cytosine deaminase enzyme that is responsible for the conversion from 5-FC into 5-FU in fungal cells [17, 18]. Some data suggest that the intestinal microbiome could be responsible for the 5-FU production and the observed side effects [19].

Despite its numerous pharmacological advantages, the use of 5-FC in clinical practice is decreasing because of the frequent occurrence of innate or acquired resistance to this drug in fungal pathogens. Thus, with few exceptions [20], 5-FC is never used as monotherapy but always in combination with another antifungal, such as amphotericin B [21, 22]. However, the elevated renal and liver toxicities of amphotericin B, that further increase 5-FC hepatotoxicity, has led to combination therapy of 5-FC more frequently with azole drugs.

**Figure 2:**
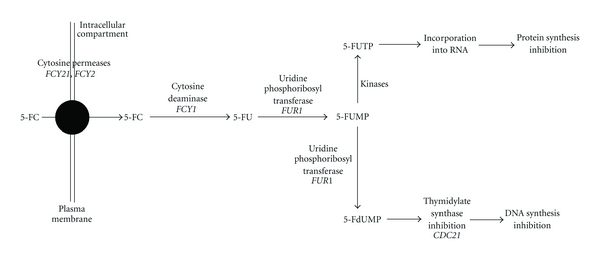
Intracellular metabolization and action mode of 5-FC in *S. cerevisiae*, adapted from [11]. In bold are indicated gene names of the respective enzymes. 5-FC: 5-fluorocytosine; 5-FU: 5-fluorouracil; 5-FUMP: 5-fluorouridine monophosphate; 5-FUTP: 5-fluorouridine triphosphate; 5-FdUMP: 5-fluoro deoxyribouridine monophosphate.

5-FC itself has no antifungal activity, and its fungistatic properties are dependent upon the conversion into 5-FU [16, 20, 23]. The drug rapidly enters the fungal cell through specific transporters, such as cytosine permeases or pyrimidine transporters [24], before it is converted into 5-FU by the cytosine deaminase [16]. 5-FU itself is converted into 5-fluorouracil monophosphate (5-FUMP) by another enzyme, uridine phosphoribosyltransferase (UPRT). 5-FUMP can then be either converted into 5-fluorouracil triphosphate, which incorporates into RNA in place of UTP and inhibits protein synthesis, or converted into 5-fluorodeoxyuridine monophosphate, which inhibits a key enzyme of DNA synthesis, the thymidylate synthase, thus inhibiting cell replication (Figure 2) [16, 25, 26].

#### 2.2.2. Polyenes

More than 200 molecules belonging to the chemical class of polyenes have an antifungal activity, most of them being produced by *Streptomyces* bacteria. However, only three possess a toxicity allowing their use in clinical practice: amphotericin B (AmB), nystatin, and natamycine.

*Streptomyces* bacteria synthesize polyenes through a gene cluster phylogenetically conserved within these species. This cluster contains genes coding for several polyketide synthases, ABC (ATP-binding cassette) transporters, cytochrome P450-dependent enzymes, and enzymes responsible for the synthesis and the binding of the mycosamine group [27]. Although it is possible to synthesize polyenes chemically, they are still produced from *Streptomyces* cultures for economic reasons.

**Figure 3:**
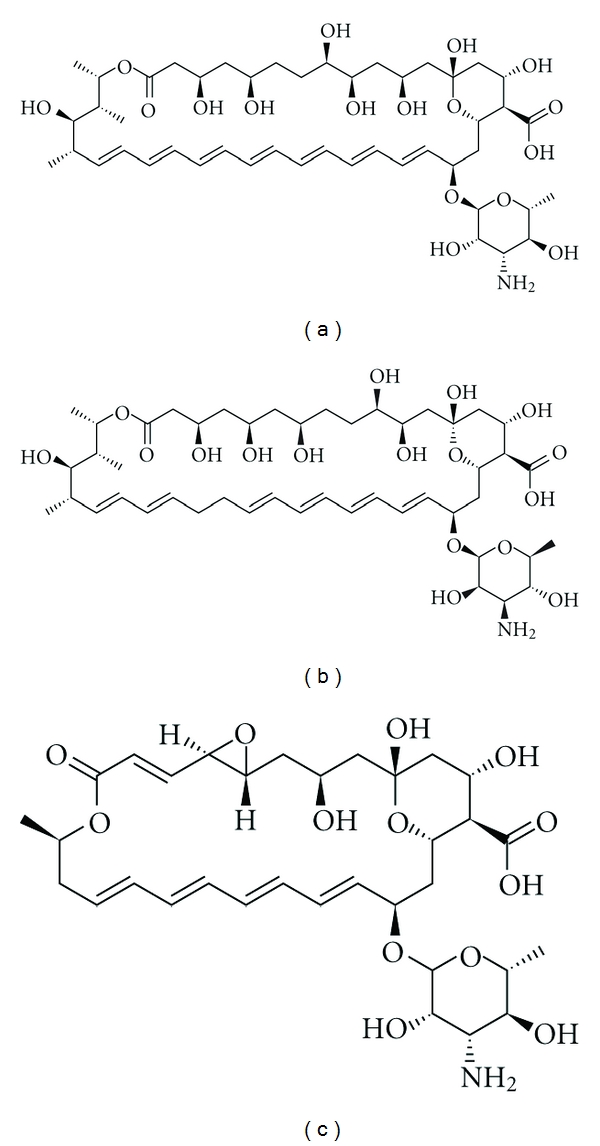
Chemical structures of amphotericin B (a), nystatin (b), and natamycin (c), three main polyene drugs.

Polyenes are cyclic amphiphilic organic molecules known as macrolides. Most of them consist of a 20 to 40 carbons macrolactone ring conjugated with a d-mycosamine group. Their amphiphilic properties are due to the presence of several conjugated doublebounds on the hydrophobic side of the macrolactone ring, and to the presence of several hydroxyl residues on the opposite, hydrophilic side (Figure 3) [28].

**Figure 4:**
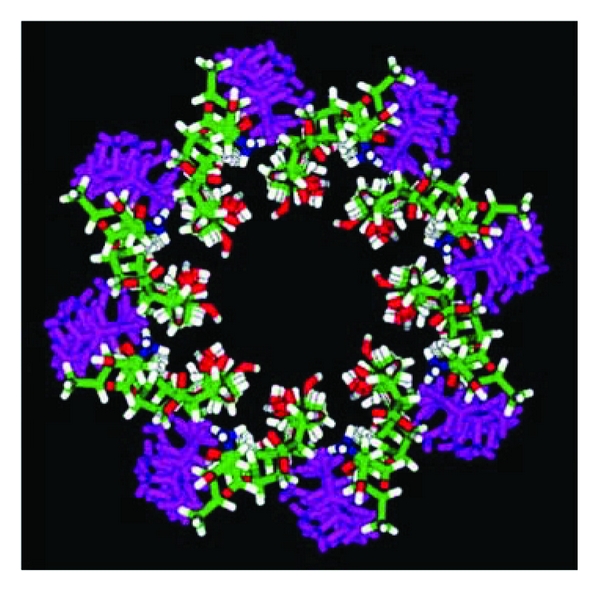
3D model of pore formed by amphotericin B into lipid bilayer of the fungal plasma membrane, adapted from Baginski et al. [29]. Amphotericin B: white (H), green (C), red (O), and blue (N); ergosterol: pink.

Polyene drugs target ergosterol, the main sterol component of fungal membranes. Their amphiphilic structure allows them to bind the lipid bilayer and form pores. Magnetic nuclear resonance data suggest that eight AmB molecules bind eight ergosterol molecules through their hydrophobic moieties, with their hydrophilic sides forming a central channel of 70–100 nm in diameter (Figure 4). Pore formation promotes plasma membrane destabilization, and channels allow leakage of intracellular components such as K^+^ ions, responsible for cell lysis [28].

While structural data suggest that polyenes target ergosterol, and despite the fact that their binding to ergosterol was experimentally demonstrated [29–31], controversy remains over a possible intracellular mode of action. Some research has suggested that polyene drugs are able to induce an oxidative stress (particularly in *C. albicans* [32, 33]) as well as their activity seems to be reduced in hypoxic conditions [34].

Polyenes possess a lower but non-negligible affinity for cholesterol, the human counterpart of ergosterol. This slight affinity for cholesterol explains the high toxicity associated with antifungals and is responsible for several side effects [28]. For this reason, only AmB is given systemically, while nystatin and natamycin are only used locally or orally. These two last molecules fortunately possess a very limited systemic activity, since their absorption trough gastrointestinal mucosa is almost nonexistent [35, 36].

For these reasons, AmB is the most used polyene antifungal for systemic infections. Due to its high hydrophobicity and poor absorption through the gastrointestinal tract, it is necessary to administer AmB intravenously [28]. However, AmB administration is accompanied with adverse effects, mostly at the level of kidneys and liver. New AmB formulations, such as liposomal AmB or lipid AmB complexes, minimize such side effects [37].

For more than 40 years, AmB was one of the goldstandards for the treatment of systemic fungal infections due to the low occurrence of acquired or innate resistance to this drug and also because of its broad range of activity [38]. Indeed, AmB is active against most yeasts and filamentous fungi. It is recommended for the treatment of infections caused by *Candida*, *Aspergillus*, *Fusarium*, *Mucor*, *Rhizopus*, *Scedosporium*, *Trichosporon*, *Cryptococcus*, and so on. AmB is also widely used to treat parasitic infections such as leishmaniasis and amibiasis [28]. Natamycin and nystatin are active against fungi belonging to the genera *Cryptococcus*, *Candida*, *Aspergillus,* and *Fusarium*. If nystatin is not used to treat molds infections, this drug is frequently used for the treatment of cutaneous, vaginal, and esophageal candidiasis, and natamycin can be used for the treatment of fungal keratosis or corneal infections [35].

#### 2.2.3. Azoles

Azoles are by far the most commonly used antifungals in clinical practice, and consequently, they are also the mostly studied by the scientific community regarding their mode of action, their pharmacological properties, and the resistance mechanisms developed by microorganisms. Azole antifungals are also largely studied by pharmaceutical companies, who seek to enhance their efficacy and to develop the *perfect antifungal*.

**Figure 5:**
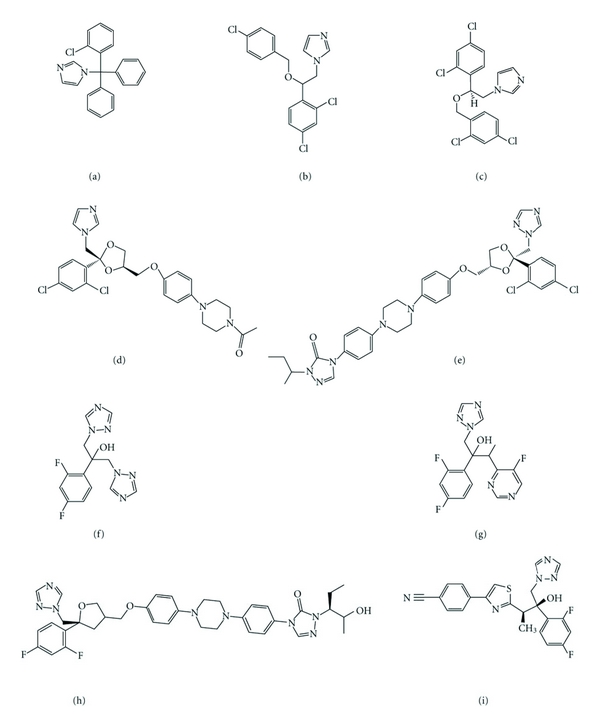
Chemical structures of the main azole antifungals, four imidazoles: clotrimazole (a), econazole (b), miconazole (c), and ketoconazole (d), two triazoles: itraconazole (e) and fluconazole (f), and three new generation triazoles: voriconazole (g), posaconazole (h), and ravuconazole (i).

Azoles are cyclic organic molecules which can be divided into two groups on the basis of the number of nitrogen atoms in the azole ring: the imidazoles contain two nitrogen atoms, and the triazoles contain three nitrogen atoms (Figure 5) [39].

Azole drugs target the ergosterol biosynthetic pathway by inhibition of a key enzyme, the lanosterol 14alpha demethylase, encoded be the *ERG11* gene. This inhibition occurs through the binding of the free nitrogen atom of the azole ring to the iron atom of the heme group of the enzyme. The resulting accumulation and metabolism of 14alpha methylated sterol species leads to the synthesis of toxic compounds, which are unable to successfully replace ergosterol [40].

The first azole was synthesized in 1944 by Woolley [41], but it was not until 1958 that scientific community began to consider azoles as potential antifungal agents. In late 1960s, clotrimazole, econazole, and miconazole became available for treatment [42]. However, their use was restricted to external application due to their high toxicity when administered orally [43, 44]. In 1968, miconazole became the first antifungal available for parenteral injection, but due to its toxicity and relatively limited range among fungal species [45], its use decreased until it was no longer commercialized.

In 1981, the Food and Drug Administration (FDA) approved a new antifungal, ketoconazole, developed by Heeres and his coworkers [46]. This drug was the only antifungal available for treatment of systemic fungal infections caused by yeasts for the following ten years. However, there are several drawbacks to this drug. It is poorly absorbed when administered orally, and no ketoconazole form has ever been developed for intravenous injection. Moreover, it cannot cross the cerebrospinal barrier and is less active in immunosuppressed patients [42, 47–49]. It causes some severe side effects such as a decrease in testosterone or glucocorticoids production and liver and gastrointestinal complications [50–52]. Lastly, numerous interactions with other drugs were described. For these reasons, the triazoles were developed.

Fluconazole became available for use by clinicians in 1990 and provided many advantages over the use of imidazoles. Fluconazole is highly hydrosoluble and therefore can be easily injected intravenously. It is almost completely absorbed through the gastrointestinal tract, and it diffuses throughout the whole body, including cerebrospinal fluid [53, 54]. Fluconazole is suitable for the treatment of superficial candidiasis (oropharyngal, esophageal, or vaginal), disseminated candidiasis, cryptococcal meningitis, coccidioidomycosis, and cutaneous candidiasis. Due to its good pharmacokinetic properties as well as its broad spectrum of activity, fluconazole was the gold-standard treatment of fungal infections during the 1990s. Unfortunately, the overprescription of this drug by physicians for prophylaxis or treatment led to an increase in resistance to azole drugs. Moreover, fluconazole is almost ineffective against most molds.

Itraconazole was approved and made available by the FDA in 1992. This triazole possesses a broad spectrum of activity across fungal species comparable to this of ketoconazole and wider than fluconazole. Moreover, it is less toxic than ketoconazole and replaced it for treatment of histoplasmosis, blastomycosis, and paracoccidioidomycosis. Contrary to fluconazole, it is also used for the treatment of infections due to species belonging to the genera *Aspergillus* and *Sporothrix* [55]. However, itraconazole is hydrophobic and is thus more toxic than fluconazole. Itraconazole is only indicated for the treatment of onychomycosis, of superficial infections, and in some cases for systemic aspergillosis [56]. A new itraconazole formulation with an enhanced absorption and a decreased toxicity was approved by FDA in 1997 [57]. An injectable formulation of itraconazole was made available in 2001 [58].

Fluconazole and itraconazole are still not the perfect antifungals, since they have some nonnegligible drug interactions with such drugs that are used in chemotherapy or with AIDS treatment. These interactions can result in a decrease in azole concentration or even to an increase in toxicity [59]. Furthermore itraconazole and fluconazole are ineffective against some emerging pathogens like *Scedosporium*, *Fusarium*, and Mucorales, and resistance to azoles is increasingly reported [60].

So-called new generation triazoles have also been developed. Voriconazole and posaconazole were approved by FDA in 2002 and 2006, respectively. Ravuconazole is currently under clinical trial phase of drug development. They possess a wide range of activity, since they are active against *Candida*, *Aspergillus*, *Fusarium*, *Penicillium*, *Scedosporium*, *Acremonium*, and *Trichosporon*, and dimorphic fungi, dermatophytes, and *Cryptococcus neoformans* [61, 62]. While new generation triazoles were shown to be more effective against *Candida* and *Aspergillus *[62], compared to classical triazoles their side effects and drug interactions are similar to those observed with fluconazole and itraconazole [63]. Likewise, fungal isolates resistant to classical triazoles are also cross-resistant to new generation triazoles.

#### 2.2.4. Echinocandins

Echinocandins constitute the only new class of antifungals made available for clinicians to fight invasive fungal infections within the past 15 years [64]. Three echinocandins were currently approved for clinical use by the FDA in United States and later by the European Agency for the Evaluation of Medicinal Products (EMEA): caspofungin in 2001 by the FDA and in 2002 by the EMEA, micafungin in 2005, and lastly anidulafungin in 2006.

**Figure 6:**
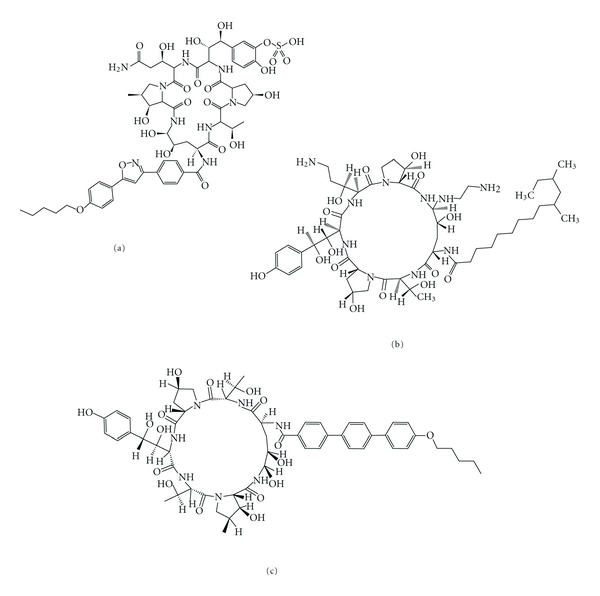
Chemical structure of the three echinocandins used in clinical practice: micafungin (a), caspofungin (b), and anidulafungin (c).

Echinocandins are synthetic derivatives of lipopeptides (Figure 6). These lipopeptides are naturally produced by several fungal species: *Aspergillus rugulovalvus* synthesizes caspofungin B, *Zalerion arboricola* synthesizes pneumocandin B, and *Papularia sphaerosperma* synthesizes papulacandin. Echinocandins are noncompetitive inhibitors of *β*(1-3)-glucan synthase, an enzyme that catalyzes the polymerization of uridine diphosphate-glucose into *β*(1-3) glucan, one of the structural components responsible for the maintenance of fungal cell-wall integrity and rigidity [65, 66]. *β*(1-3)-glucan synthase consists of an activating and a catalytic subunit encoded by *FKS* genes. In most fungi, two *FKS* genes are found within the genome. It has been shown in the model organism *Saccharomyces cerevisiae* that *FKS1* is expressed during the vegetative growth phase and *FKS2* during sporulation [67]. Echinocandins are able to inhibit both isoforms of the enzyme [68]. Inhibition of *β*(1-3)-glucan synthase leads to cell wall destabilization and to the leakage of intracellular components, resulting in fungal cell lysis [69].

These drugs are poorly absorbed in the gastrointestinal tract because of their high molecular weights and are therefore only used intravenously. Their pharmacologic properties are one of the reasons responsible for the approval of echinocandins by the FDA and the EMEA. These molecules possess a low toxicity (very rare side effects were reported) and are slowly degraded, and a daily injection is sufficient, and contrary to other antifungals, interactions between echinocandins and other drugs are rare [64]. Combined therapy between echinocandins and AmB or another azole often leads to a synergistic effect or at least to an additive effect [70, 71].

Another reason for which the echinocandins were approved is their activity spectrum. Indeed, echinocandins are active against most fungal species, including *Candida* and *Aspergillus*. For still unclear reasons, these molecules are fungicidal in *Candida* but only fungistatic in *Aspergillus* [72, 73]. Moreover, fungicidal activity of echinocandins is species and isolate dependent within the *Candida* genus [74]. There exist several species within the fungal kingdom for which the echinocandins are ineffective. Such species include *Cryptococcus neoformans* [75] or species belonging to *Trichophyton* and *Fusarium* genera. Other species have an intermediate susceptibility to echinocandins, such as *Scedosporium apiospermum*, *S. prolificans*, and *Cladophialophora bantiana *[72]. However, echinocandins constitute a good alternative to fight against fungal infections and most of treatment of infections for which classical therapy with azoles or polyenes failed are successfully managed with echinocandins [64]. Therefore, caspofungin is indicated for the treatment of candidemia and invasive candidiasis, for fungal infection prophylaxis, and for the treatment of invasive aspergillosis for which itraconazole, voriconazole, or AmB is ineffective. Micafungin is used for treatment of candidemia and is particularly indicated for fungal infection prophylaxis in bone-marrow transplant patients. Anidulafungin has no particular indications, but its main advantage is its slow degradation in the body without liver or kidney involvement, thus it can be used in patients with liver and/or kidney insufficiencies [76].

**Figure 7:**
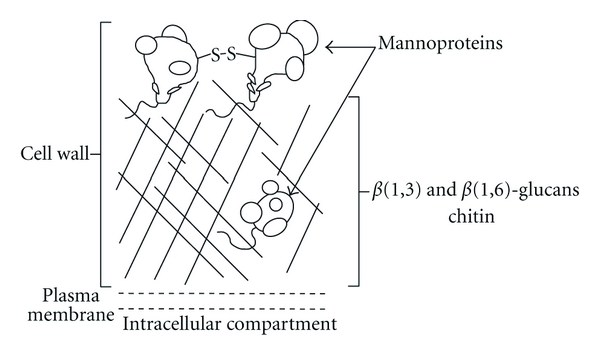
Schematic representation of *S. cerevisiae* cell wall, adapted from Stone et al. [69].

What makes echinocandins unique is their fungal target. For many years, the fungal cell wall was considered to be a promising target for the development of new antifungal molecules [68]. The fungal cell wall contains elements that have no equivalents in human [77]. Its integrity is necessary for the fungal survival, since it provides a physical barrier against the host immune cells or against other microorganisms. Cell wall integrity is also responsible for osmolarity homeostasis and the maintenance of cell shape and size. Cell wall is also indispensable to essential enzymatic reactions and as an important role in cell-cell communication. The internal layer of the cell wall is composed of a *β*(1-3)-glucans and chitin web, in which are included some mannoproteins, while external layer is composed of mannoproteins (Figure 7) [77].

#### 2.2.5. Other Antifungal Agents

Considering that the ergosterol biosynthetic pathway requires several enzymes that are unique to fungi, they constitute good targets for antifungal therapy, and three minor ergosterol biosynthesis inhibitors are used as topical antifungals. The allylamines and thiocarbamates, such as terbinafine and tolnaftate, both inhibit the *ERG1*-encoded enzyme, squalene epoxidase. The morpholines such as amorolfine act by inhibiting two different enzymes of the pathway, the Δ7,8-isomerase (encoded by *ERG24*) and the C14-reductase (encoded by *ERG2*). Despite their wide spectrum of activity, these antifungal agents are essentially used to treat dermatophyte infections such as *tinea capitis*, *tinea pedis*, and onychomycosis, because they do present numerous side effects.

Ciclopirox is also used as a topical antifungal agent, but its mode of action remains poorly understood in fungi [78, 79]. Another drug, griseofulvin, inhibits mitosis by interfering with microtubules function [80].

### 2.3. Incidence of *In Vitro* Resistance in Fungal Infection

The incidence of fungal infections has drastically increased over the past three decades and was simultaneously accompanied by increased acquired and innate resistance to antifungal drugs. However, antifungal resistance occurrence has to be considered independently for each antifungal class and for each fungal genus. Moreover, epidemiological data regarding incidence of resistance among fungal species is not identically distributed worldwide [81–83]. Lastly, clinical resistance, defined as the treatment failure in the patient, does not always correlate with *in vitro* resistance, measured as an increase in minimal inhibitory concentration of a drug. In this paper, only *in vitro* resistance incidence will be described.

#### 2.3.1. 5-Fluorocytosine

5FC resistance is a very common phenomenon [9, 16, 84]. The development of resistance can be intrinsic, as is the case for *C. tropicalis*, or acquired through the selection of resistant mutants after antifungal exposure. Within the *Candida* genus, 7% to 8% of clinical isolates are resistant to 5FC, and this frequency increases to 22% when only non*albicans Candida* species are considered. One to two percent of *Cryptococcus neoformans* clinical isolates are resistant to 5FC [85]. Filamentous fungi such as *Aspergillus* and dermatophytes are not susceptible to 5FC.

#### 2.3.2. Polyenes

Despite the reported increase of polyene resistance, it remains a relatively rare event in clinical isolates of fungal pathogens [86], probably in relation with their mode of action, and the absence of systematic and standardized determination of susceptibility of clinical isolates [87]. The incidence of strains resistant to polyenes may thus be largely underestimated. Most fungal species are considered as susceptible to polyene drugs. However, some of them are intrinsically poorly susceptible to these antifungals, such as *C. glabrata*, *Scedosporium prolificans,* or *Aspergillus terreus* [38]. Some species are more prone to acquire polyene resistance. Among yeasts, one may cite *C. lusitaniae* [88, 89], *C. guilliermondii* [88], *C. krusei* [38], and *Trichosporon beigelii* and among filamentous fungi *Scedosporium apiospermum* and *Sporothrix schenckii* [90, 91].

#### 2.3.3. Azoles

The early 1990s was the start of a drastic increase in resistance among fungal clinical isolates. However, the improvement of antifungal therapeutic strategies throughout the last several years has helped to stabilize resistance frequencies. Increase in azoles use selected less susceptible species as well as those able to develop resistance. This led to a shift in the pathogenic fungal species encountered in clinic.

#### 2.3.4. Echinocandins

Echinocandins resistance is a rare event [92]. For example, it is estimated that more than 97% of clinical isolates belonging to the *Candida* genus are susceptible to these drugs [93, 94]. Contrary to acquired resistance in other fungi, intrinsic echinocandin resistance in *Cryptococcus neoformans* is not linked with a *FKS1* or *FKS2* mutation. Indeed, *C. neoformans* *β*(1–3)-glucan synthase is inhibited by echinocandins, but this yeast is able to grow in the presence of high concentrations of these drugs. *C. neoformans* resistance to echinocandins seems to be due to a particular cell-wall polysaccharides composition in this species [95].

#### 2.3.5. Incidence of *In Vitro* Resistance on Patient Care

As antifungal *in vitro* resistance poorly correlates with clinical outcome, better attention was needed to define parameters that produced reproducible and reliable intra- and interlaboratory results. For this purpose, two standardized methods for the testing of yeast and mould isolates (CLSI and Eucast) are recognized as the gold standards for drug susceptibility testing [96–98]. These standardized approaches produce susceptibility results comparable between laboratories, which may help to establish breakpoints for antifungal agents (see [96–98] for details). These breakpoints, defined as susceptibility ranges, together with pharmacokinetic and pharmacodynamic analyses and identification of resistance mechanisms, help to assess the *in vivo* activity of antifungal agents in invasive disease and therefore clinical outcome [99, 100].

## 3. Drug Resistance Molecular Mechanisms

**Figure 8:**
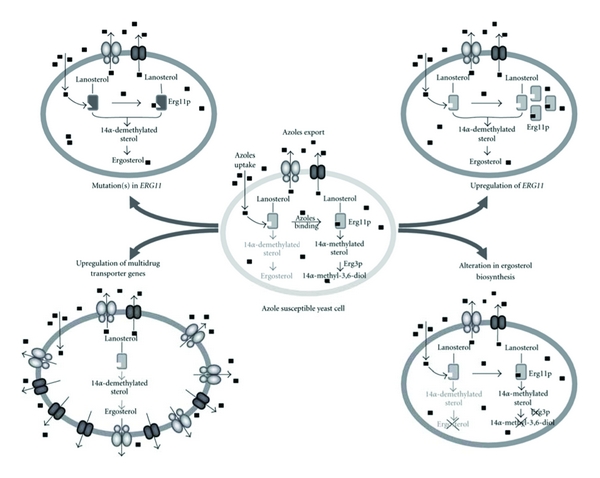
Mechanisms of resistance to azole compounds in *C. albicans*.

Microorganisms develop mechanisms to counteract the fungicidal or fungistatic effects of all antifungals classes that are based on three major mechanisms, namely, (i) reducing the accumulation of the drug within the fungal cell, (ii) decreasing the affinity of the drug for its target, and (iii) modifications of metabolism to counterbalance the drug effect (Table 2). The molecular mechanisms leading to azole resistance have been most studied in yeast, and taking them as an example, such mechanisms are divided into four categories (Figure 8) [101]: (i) decrease in azole affinity for their target, (ii) increase in azole target copy number, (iii) alteration of ergosterol biosynthetic pathway after azoles action, and (iv) decrease in intracellular azole accumulation. In some highly resistant clinical isolates, sampled from long-term treated patients, several mechanisms of resistance are often combined [102, 103]. This increase in resistance along antifungal treatment is due to the sequential acquisition of different mechanisms [104–106]. In the following section, the molecular basis of the resistance mechanisms to antifungals will be described. 

### 3.1. Increase of Drug Efflux

#### 3.1.1. ABC Transporters

*CDR1 *and *CDR2 *(*Candida* drug resistance 1 and 2) from *C. albicans *are the two major ABC transporters involved in azole resistance in this species*. CDR1* and *CDR2* can be coordinately upregulated in some azole-resistant strains or by exposure to a wide variety of chemically unrelated inducers such as terbinafine, amorolfine, fluphenazine, or steroid hormones. Several *cis*-acting regulatory elements responsible for the regulation of these two genes were identified by several investigators [107–111]. Promoter deletion studies have revealed 5 different regulatory elements in the *CDR1 *promoter including a BEE (basal expression element), a DRE (drug responsive element), two SREs (steroid responsive element), and a NRE (negative regulatory element) (see Table 3 for details). Internal deletions of the BEE and DRE motifs in the *CDR1* promoter affect basal *CDR1* expression and drug-induced expression, respectively [107]. SRE1 and SRE2 were reported to be involved in the response to steroid hormones: with SRE1 responding only to progesterone and SRE2 to both progesterone and *β*-oestradiol [108]. Finally, the deletion of the NRE motif leads to an increase in the basal expression of *CDR1* [110, 111]. In contrast to *CDR1*, the *CDR2* promoter contains only a DRE motif (Table 3) [107]. Among these different *cis-*acting elements, DRE was the only element involved in constitutive high expression and in transient upregulation of both *CDR1 *and *CDR2*. This DRE sequence was functionally analyzed by systematic mutation each base of the initially described DRE sequence [107, 112]. The data obtained from systematic mutational studies are in agreement with ChIP-Chip assays performed with the *trans-*acting factor binding to the DRE [113]. In other *Candida* species, functional homologues to *CDR1* and *CDR2* were described as involved in drug resistance. In *C. glabrata*, *CgCDR1* and *CgCDR2* (formerly denoted *PDH1*) as well as *SNQ2* (another ABC transporter coregulated with *CgCDR1* and *CgCDR2*) are upregulated in azole-resistant clinical isolates and participate in azole resistance [114–118]. All the three genes contain *cis-*acting elements in their promoters, so-called PDRE. These elements are similar to those described in *S. cerevisiae* for *PDR5*, an ABC transporter involved in drug resistance of *S. cerevisiae* [119, 120]. Disruptions of *CgCDR1* and *CgCDR2* lead to hypersusceptibility to fluconazole, cycloheximide, and chloramphenicol [115, 117]. In both *C. albicans* and *C. glabrata*, *CDR1* was shown to be the main contributor in azole-resistance among the ABC-transporters [121–123]. Other ABC-transporters from *C. dubliniensis *(*CdCDR1* and *CdCDR2*) [124, 125], *C. krusei* (*ABC1* and *2*) [126, 127], *C. tropicalis *(*CDR1-*homologue), and *C. neoformans* (*CnAFR1*, AntiFungal Resistance 1) were reportedly upregulated in azole-resistant isolates. In *A. fumigatus*, *atrF,* and *AfuMDR4* are upregulated in itraconazole-resistant strains [128–130]. The *cis-*acting regulatory elements of these genes are still awaiting in-depth dissection analysis. The overexpression of ABC-transporters have also been identified as a resistance mechanism to azole in *Aspergillus nidulans* [131, 132].

**Figure 9:**
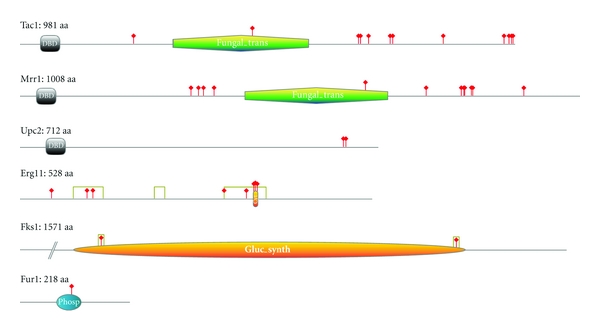
Point mutations affecting antifungal susceptibility in clinical isolates of *C. albicans*. Indicated functional domains were determined using either Prosite or Pfam tools. Only mutations which involvement in antifungal resistance was experimentally demonstrated are indicated by a red stick. Hot spot mutations in Erg11 and Fks1 are delimitated by gray boxes (Point mutation localization references: Tac1 [112], Mrr1 [133], Upc2 [134], Erg11 [135], Fks1 [136], Fur1 [137]). Drawings of the proteins were made with Prosite My Domain-Image Creator tool.

The identification of *trans-*acting factors regulating ABC-transporters in pathogenic fungi relied first on the well-described *S. cerevisiae *PDR network as a model [138–142]. Since the Zn2-Cys6 transcription factors *PDR1/PDR3* are master regulators of this network in *S. cerevisiae*, an *in silico* search for *PDR1/PDR3* homologues in fungal genomes was performed. Data so far available found only one functional homologue in *C. glabrata *[120]. CgPdr1p has 40% and 35% identity with Pdr1p and Pdr3p, respectively [143], and was able to complement a *pdr1*Δ *S. cerevisiae* mutant strain. Likewise, *PDR1* deletion in *C. glabrata* leads to a loss of *CgCDR1* and *CgCDR2* regulation and to a sharp decrease in azole MICs. [144]. Three studies have identified separate gain-of-function mutations in *CgPDR1* alleles of azole-resistant strains which are responsible for constitutive high expression of *CgCDR1*, *CgCDR2*, *SNQ2*, and *CgPDR1* itself (Figure 9) [120, 145, 146].

Attempts to identify *C. albicans PDR1/3* functional homologues were undertaken to complement the absence of *PDR1/PDR3* in *S. cerevisiae* by genetic screens. Several genes were identified including *FCR1* and *FCR3* (FluConazol Resistance) [147–149] and *SHY1*-3 (Suppressor of Hypersusceptibility) [150] (formerly, resp., named *CTA4, ASG1 *and* ATF1*). *FCR1*, *CTA4, ASG1*, and* ATF1* encode Zn2-Cys6 transcription factors, while *FCR3* encodes a bZip transcription factor. Even though *FCR1* was able to restore *PDR5 *expression in a *pdr1*Δ/*pdr3*Δ *S. cerevisiae* mutant strain, its disruption in *C. albicans* resulted in decreased susceptibility to fluconazole, suggesting that *FCR1* acts as a negative regulator of fluconazole susceptibility [147]. Nevertheless, the target genes of *FCR1* in *C. albicans* are not yet known. Up to now, the relevance of *FCR3* in azole resistance has not been addressed in *C. albicans*. *CTA4, ASG1*, and* ATF1 *expression in *S. cerevisiae *could restore *PDR1/PDR3* functions in *S. cerevisiae*; however, their disruption in *C. albicans* did not affect azole susceptibility and expression of *CDR1* and *CDR2 *[150]. An additional regulator of *CDR1* was identified by a genetic screen in *S. cerevisiae* with a *LacZ *reporter system under the control of the *CDR1* promoter. A *C. albicans* gene was subsequently identified that encodes for a protein CaNdt80p similar to the *S. cerevisiae* meiosis specific transcription factor Ndt80p. Disruption of *CaNDT80* in *C. albicans* was shown to affect basal expression levels of *CDR1* in *C. albicans* and reduce the ability of this gene to be upregulated in the presence of miconazole [151, 152]. More recently, Ndt80p was shown to have a global effect on azole-resistance through is regulon which includes many genes involved in ergosterol metabolism [153].

The release of the entire data from the *C. albicans* genome sequence has encouraged other approaches for identifying *trans-*regulators of *CDR1* and *CDR2*. Since the DRE motifs present in the promoter of *CDR* genes contains two CGG triplets that are potentially recognized by Zn2-Cys6 transcription factors (TF) [154–157], it was likely that one of the 78 ORFs encoding proteins with Zn2-Cys6 signatures could be involved in the regulation of *CDR1* and *CDR2*. Interestingly, genome data revealed that three of these ORF (the so-called “zinc cluster”) were located in tandem close to the mating type locus (*MTL*) at a distance of 14 kb [158]. Homozygosity at the* MTL* locus is associated with the development of azole resistance in *C. albicans *[159], thus indicating that one the genes of the zinc cluster could control *CDR1* and *CDR2 *expression. As a matter of fact, deletion of one of these Zn2-Cys6 TF-encoding genes in an azole-susceptible strain led to increased drug susceptibility and loss of transient *CDR1* and *CDR2 *upregulation in the presence of inducers. This gene was named *TAC1* for transcriptional activator of *CDR* genes [158]. Consistent with the mutant phenotype, Tac1p can bind *in vitro* and *in vivo* to the DRE [112, 158]. However, *TAC1* is not involved is the basal expression of *CDR1* controlled at least by the BEE [158]. Hyperactive alleles that confer constitutive high* CDR1* and *CDR2 *expression, and therefore drug resistance to a *tac1*Δ*/*Δ mutant strain of *TAC1*, were isolated from azole-resistant strains. Wild-type and hyperactive alleles differed by point mutations defined as gain-of-function mutations (GOF). Up to now, at least 15 GOF were described in *TAC1* at 12 different positions [112, 158, 160, 161] (Figure 9). Wild-type and hyperactive alleles are co-dominant for the expression of their phenotypes [112, 158, 160, 161], and because of this property, high drug resistance levels correlate with homozygosity of hyperactive alleles. Interestingly, the *TAC1* locus and the associated *MTL* are rendered homozygous in the development of azole resistance. Such events are accomplished by rearrangements on chromosome 5 including mitotic recombinations on one chromosome 5 arm or the loss of one chromosome 5 homologue followed by duplication [160]. Increase of resistance can still be obtained by isochromosome formation with the left arm of the chromosome 5. This allows for the increase of drug resistance genes present on this chromosome (among which *TAC1* and *ERG11*) and thus can contribute to drug resistance increase [160–164]. Up to this date, regulation of Tac1p activity remains unknown.

#### 3.1.2. Major Facilitator Superfamily (MFS) Transporters

In *C. albicans*, *MDR1* (MultiDrug Resistance 1, previously named *BEN^r^*for Benomyl resistance) is a transporter currently shown to be the only MFS transporter involved in azole resistance of clinical isolates [165, 166]. *MDR1* is not usually expressed at detectable levels in fluconazole-susceptible isolates, but is constitutively upregulated in some fluconazole-resistant isolates. As for *CDR1* and *CDR2*, *MDR1* can be specifically transiently upregulated by drugs such as benomyl, cycloheximide, methotrexate, and several oxidizing agents [165]. MFS transporters are known to be involved in azole resistance of other fungal species. Homologues of *MDR1* in *C. dubliniensis *and* C. tropicalis*, named *CdMDR1* and *CtMDR1*, respectively, are upregulated in azole-resistant strains [167–170]. In *A. fumigatus*, *in vitro*-generated itraconazole-resistant isolates show constitutive high expression level of the MFS transporter, AfuMDR3 [128]. The role of *cis-*acting regulatory elements in resistance was investigated in the *C. albicans MDR1 *gene by separate studies. Two of the studies undertaken by Rognon et al. and Riggle and Kumamoto identified a similar region, called BRE (benomyl response element) or MDRE (*MDR1* drug resistance element) respectively. This region is responsible for the constitutive high expression of *MDR1 *in fluconazole-resistant isolates [171, 172] and was also shown to be responsible for the response to benomyl [172]. A second regulatory element involved in the response of *MDR1 *to oxidative stress is designated HRE (H_2_O_2_ response Element). This region contains two YRE (*YAP1* response element) motifs [173], one perfectly conserved (-532 TTAGTAA-526) and the other with two mismatches (-549 TAACTAT-543). Interestingly, the HRE is not required for constitutive upregulation of *MDR1 *in azole-resistant isolates. A separate study undertaken by Hiller et al. described three distinct *cis-*activating regions (1, 2, and 3) in *MDR1*. Region 2, which overlaps with encompassing the HRE, was implicated in benomyl-dependent *MDR1* response [174]. Region 1 and 3, close to the BRE/MDRE region, were required for a constitutive high expression of *MDR1* in an azole-resistant isolate.

*MDR1* expression was shown to be regulated by at least four *trans-*acting factors: Cap1p [175–177], Mrr1p [178, 179], Upc2p [180, 181], and Mcm1p [182]. Nucleotide sequence data of *cis-*acting elements has provided some clues to their identification. When comparing the *MDR1 cis-*acting elements with existing transcription factor binding site databases, several putative *trans-*acting elements were identified. As mentioned above, the HRE of *MDR1* contains Yap1p binding sites. The bZip transcription factor Cap1 was shown to directly interact with the *cis-*acting domains [177] and to be involved in drug resistance [175]. The BRE/MDRE motif contains a perfect match for the Mads-box transcription factor Mcm1p in its sequence. Mogavero et al. showed that Mcm1p acts as a coregulator for Cap1 and Mrr1p and is not required for *MDR1* upregulation by H_2_O_2_ but is required for full *MDR1* induction by benomyl [182]. Genome-wide transcriptional analyses of clinical isolates that exhibit *MDR1*upregulation permitted the identification of a Zn2-Cys6 transcription factor that is coregulated with *MDR1* [179]. Deletion of *MRR1* in azole-resistant strains abolishes the constitutive overexpression of *MDR1*, therefore identifying Mrr1p as a central regulator of *MDR1*. Like for *TAC1*, two types of alleles were distinguished for *MRR1*. Wild-type alleles are needed for a transient upregulation of *MDR1* by drug exposure. In contrast, hyperactive alleles confer constitutive overexpression of *MDR1* and therefore also confer increased resistance to fluconazole [179]. Wild-type and hyperactive alleles differ by GOF mutations and until now, 14 GOF mutations at 13 positions were described in hyperactive Mrr1 [180] (Figure 9). Interestingly, hyperactive Mrr1 proteins were also shown to be able to confer Mdr1p-independent drug resistance probably through the regulation of oxydoreductases implicated in the detoxification of yeast cells after fluconazole exposure [183]. A blast analysis in *C. dubliniensis* allowed for the identification of a gene encoding for a protein that shares 91% of identity with Mrr1 of *C. albicans* [133] and able to complement a *CaMrr1*Δ mutant strain. The properties of CdMrr1 are similar to those of CaMrr1 and two types of alleles can also be distinguished. Until now 5 GOF mutations were identified and analyzed in hyperactive CdMrr1 proteins [133].

### 3.2. Target Alteration

#### 3.2.1. Target Mutation

Another mechanism by which fungal pathogens are able to develop resistance is a decrease in antifungal affinity for their respective targets, without a major decrease in target activity. Such is the case for azole drugs, in which a decreased affinity between azole and a mutated lanosterol 14*α*-demethylase, can lead to resistance. A point mutation in the *ERG11* gene that codes for lanosterol 14*α*-demethylase leads to the complete inhibition of the binding capacity of the azole drug to its target [184, 185]. Numerous of these point mutations identified in *ERG11* were previously described, and their involvement in azole resistance was experimentally demonstrated for fungi such as *Cryptococcus neoformans* [186], *C. albicans* [187], (see also Figure 9), and *C. tropicalis* [167]. In *Aspergillus fumigates, CYP51A* and *CYP51B* encode two distinct forms of 14*α*-demethylase and mutations in the first of these two genes seem to be the most frequent mechanism responsible for azole resistance in clinical isolates. In this species, it was demonstrated that the nature of the nucleotide mutation, and therefore, the nature of the amino acid substitution, influences the development of resistance to different azole agents [188–192]. Interestingly, it was demonstrated that some clinical isolates share common mutations in Cyp51A with environmental azole-resistant strains, suggesting that some clinical azole resistant isolates might originate from the environment [193–195].

While target site alteration is far from being the most significant mechanism of resistance to azole drugs, it is the only known mechanism by which fungal pathogens are able to develop resistance to echinocandin drugs. This was demonstrated for *S. cerevisiae* and *C. albicans*. Echinocandin resistance is systematically associated with point mutations in either* FKS1* or *FKS2* [196, 197]. Analysis of the location of these mutations within the *FKS* genes led to the characterization of two regions, the so-called “hot spots”, integrity of which seems to be essential for enzyme activity [136]. In contrast to azoles and *ERG11*, mutations in *FKS1* did not alter the *β*-glucane synthase affinity for its target but decreased only the enzyme processivity [198]. Hot-spot mutations have also been identified in other species, such as *C. glabrata* [196, 199], *C. krusei* [200], *Scedosporium apiospermum* [201], and *A. fumigatus* [202, 203] (Figure 9).

Numerous enzymes of the pyrimidine salvage pathway are involved in 5FC mode of action and thus numerous molecular mechanisms could lead to resistance to this drug [16, 204]. The most frequently found mechanism in clinical isolates of pathogenic fungi is a point mutation in the *FUR1* gene that encodes the enzyme responsible for the conversion of 5FU into metabolites able to enter the cytosine metabolism (Figure 9) [14]. *FUR1* mutation leads to complete resistance to both 5FC and 5FU in fungi. A second, frequently reported mutation leads only to resistance of 5FC. This second mutation is a point mutation in the *FCY1* gene that codes for cytosine deaminase, the enzyme responsible for the conversion from 5FC into 5FU. Several such point mutations that lead to decreased activity of the cytosine deaminase were identified, essentially in *Candida* yeast species such as *C. glabrata* [205, 206] and in *S. cerevisiae* [207].

#### 3.2.2. Target Expression Deregulation

A third mechanism of drug resistance is the deregulation of the drug target expression. For drugs targeting, the biosynthesis of ergosterol, such as azoles, terbinafine, or fenpropimorph, even relative short exposures of two to three hours lead to the transient upregulation of the *ERG* gene family in *C. albicans, glabrata, tropicalis*, and* krusei* [208]. These data suggest a common regulation of the ergosterol biosynthetic pathway in the presence of inhibitors. Longer azoles* in vitro* exposures (minimum 24 h) lead to constitutive upregulation of *ERG* genes, including *ERG11* [209], and decrease drug susceptibility. In clinical isolates of *C. albicans* and *C. dubliniensis *resistant to azoles isolated from HIV patients, upregulation of *ERG11* was described as a minor mechanism often combined with other more major mechanisms of resistance such as pump overexpression or *ERG11* mutations [103, 169, 210]. The overexpression of *ERG11* originates either by gene dosage effect through duplication of the gene or by upregulation of the gene by a *trans-*acting factors, both hypotheses were verified. In *C. albicans* and *C. glabrata*, it was shown that increased azole resistance due to *ERG11* upregulation was in fact due to genome rearrangement via formation of an isochromosome in *C. albicans* and duplication of a chromosome in *C. glabrata*, and therefore amplification of *ERG11* [163, 211]. In *Cryptococcus neoformans,* the well-known *SRE1* gene was shown to regulate the ergosterol biosynthesis pathway and also to be involved with virulence of the fungus [212]. In *S. cerevisiae*, the *ERG* gene family was shown to be regulated by two zinc cluster transcription factors encoded by *ScUPC2* and *ScECM22*. Two homologues of *ScUPC2* were found in the genome of *C. glabrata*: *CgUPC2A* and *CgUPC2B*. It appears that while both transcription factors regulate sterol biosynthesis and exogenous uptake, only CgUpc2A is responsible for the regulation of the transcription of the *ERG* gene family in response to sterol inhibitors [213]. In *C. albicans*, only one gene homologue of *ScUPC2*/*ScECM22* was found and named *CaUPC2* [214, 215]. It was shown that CaUpc2 is necessary for the upregulation of *ERG* genes in the presence of ergosterol synthesis inhibitors. Moreover, the *upc2* Δ/Δ mutant shows increased susceptibility to most drugs and a decrease in sterol uptake as compared to the wild-type strain [214, 215]. Further studies demonstrated the ability of CaUpc2 to bind to the ARE motif in the promoter of *C. albicans ERG11 *(Table 3) [215, 216]. Genome-wide location analysis of CaUpc2 confirmed the SRE motif as the DNA binding site, and also confirmed the *ERG* gene family as a CaUpc2 target as well as *CDR1*, *MDR1*, and *UPC2* itself as new target genes. Analysis of clinical strains resistant to fluconazole with upregulated *ERG11* expression, demonstrated the existence of a hyperactive allele of *CaUPC2* that confers intrinsic upregulation of *ERG* genes. Currently, two GOF mutations were described for CaUpc2 (Figure 9) [134, 180].

### 3.3. Metabolism Modification

#### 3.3.1. Echinocandins Paradoxical Effect

Some yeasts and filamentous fungi are able to grow in elevated echinocandin concentrations much higher than the MICs [136, 217]. This phenomenon, called paradoxical effect, is due to the metabolic adaptation of microorganism and is mediated by the cell wall integrity signalization pathway. This response is the direct consequence of the *β*(1-3)-glucans synthesis inhibition and the subsequent cell wall composition modifications, upon echinocandin administration [218, 219]. Several studies suggest that the magnitude of the paradoxical effect is variable depending on the microorganism itself as well as on the echinocandin nature. Therefore, the paradoxical effect would be more pronounced in the presence of caspofungin as compared to anidulafungin or micafungin [220]. However, the clinical significance of paradoxical effect has never been studied nor has it ever been observed in echinocandin-treated patients [86].

#### 3.3.2. *De Novo* Synthesis of Pyrimidines

It is possible that 5FC resistance could be the consequence of an overall induction of the *de novo* pyrimidine biosynthetic pathway. In this case, the antifungal drug competes with the regular pyrimidine intermediate metabolites for incorporation into nucleic acids [16]. This increase in activity of the *de novo* pyrimidine biosynthetic pathway is reflected by an increased expression of the *CDC21* gene, whose product is inhibited by 5FC [205]. *FUR1* mutations could lead to 5FC resistance. However, its downregulation has also been demonstrated to be involved in 5FC decreased susceptibility. A 4-fold decreased expression of this gene of high importance in 5FC mode of action is sufficient to lead to a total resistance to this pyrimidine fluorinated analog in *C. glabrata* [84].

#### 3.3.3. Ergosterol Biosynthesis Pathway Alteration

Modifications of main metabolic pathways could also lead to azole drugs resistance. For example, alteration of the late steps of the ergosterol biosynthetic pathway through inactivation of the *ERG3* gene gives rise to cross-resistance to all azole drugs [101]. Indeed, the antifungal activity of azole drugs relies on the synthesis of toxic 14*α* methylated sterols by the late enzymes of this pathway. A point mutation that occurs in the *ERG3* gene can lead to the total inactivation of C5 sterol desaturase. In this case, toxic 14*α* methylated sterols are no longer synthesized and even in the presence of azole drugs sterols species able to replace ergosterol are generated. While very uncommon, this mechanism was identified in several clinical isolates of *C. albicans *[221–223].

#### 3.3.4. Plasma Membrane Composition Variation

Polyene drugs do not require internalization into fungal cells in order to exert their antifungal activity, since they incorporate into the plasma membrane from the external side. Thus, they escape metabolizing enzymes and efflux systems, and the only possibility for fungi to develop resistance to polyene is to modify their target, ergosterol. However, ergosterol is responsible for the integrity and fluidity of the plasma membrane, and therefore, possibilities to compensate for its absence are very limited. Although rarely described, resistance mechanisms responsible for acquired or innate resistance to polyene drugs were studied in several fungal species. In each case, resistance to polyenes results from a decrease or total absence of ergosterol in the plasma membrane through mutations in nonessential genes of the ergosterol biosynthetic pathway [224]. Molecular polyene resistance mechanisms were described in laboratory mutants of yeasts belonging to the *Candida* genus and in *S. cerevisiae*. Thus, both *ERG11* deletion in *C. albicans* [225] and *ERG3* deletion in *S. cerevisiae* [226] lead to mutants with cross-resistance to azole and polyene drugs. Likewise, *ERG6* inactivation in *C. lusitaniae* [227] and *S. cerevisiae* results in polyene resistance [228]. Regarding clinical isolates, very few data is available. Only a few studies have associated polyene resistance to an *ERG3* mutation in clinical isolates of *C. albicans* [221, 229] and to an *ERG6* mutation in *C. glabrata *[230, 231].

#### 3.3.5. Biofilms

“United we stand, divided we fall”. This statement is certainly true concerning the fight between fungi and antifungals. It is well characterized that microbial communities engulfed in a polysaccharides-rich extracellular matrix, also known as biofilm, are by far more resistant to antifungal drugs than isolated cells. Fortunately, few pathogenic species within the fungal kingdom are able to form biofilms. The mostly known and widely studied of those species able to form biofilms are the species of the *Candida* genus [232]. Another yeast frequently responsible for biofilm-associated infections is *Cryptococcus neoformans* [233, 234]. Some clinical cases have also reported the involvement of other yeast species, such as *Pichia fabianii* [235] or *Trichosporon asahii* [236]. Additionally, it is now accepted that filamentous fungi, and particularly those of the *Aspergillus* genus, can grow as biofilms in humans [237–239]. Fungal biofilms are frequently polymicrobial biofilms, meaning that bacterial species frequently associate with one or several fungi [240, 241]. In medical mycology, biofilms constitute a real concern in the fields of invasive and dental medicine. They constitute a nonnegligible source of nosocomial fungal infections, essentially through the use medical devices. Moreover fungal biofilms are resistant to almost all the currently used antifungals, with the exceptions of echinocandins and lipid formulations of AmB [242]. The molecular mechanisms underlying the persistence of the fungal biofilms despite antifungal treatment remain unclear. It is likely that biofilm resistance is the result of a combination a multiple factors, among them an increased expression of efflux pumps, a modification of plasma membrane composition, and the biofilm-produced extracellular matrix itself [232, 243].

## 4. Development of New Antifungal Strategies

Current antifungal treatments are limited in their capacity to treat infections, especially systemic infections and no considerable advancements in antifungal therapies were developed recently. New therapies are therefore needed against pathogenic fungi. Several approaches were developed during the last several years in order to find new solutions. Researchers aim to discover new antifungal drugs either by testing already existing medical compounds, compounds from natural sources such as plants, sea, microorganisms or by systematic screens of chemical compound libraries. Researchers also strive to elucidate the underlying biology of fungal microorganism both *in vitro* and *in vivo*. Host-fungal interactions play a critical role for all fungal pathogens. Targeting this interaction provides novel therapies, which could be used alone or in combination with existing antifungal drugs. Such a combination may also determine the development of antifungal drug resistance.

### 4.1. Development of New Antifungal Active Compounds

Much effort has gone towards analyzing the antifungal properties of what is called natural compounds (NP) or natural bioactive compounds isolated from plants, other microorganisms, or marine organisms [244–246]. Some such compounds are investigated because their known triggering mechanisms important for fungi, while other compounds are tested blindly for their antifungal properties. Currently, none of these studies have produced a compound suitable for the clinical trial stage although interesting results were obtained.

Other studies focused on *in vitro* screens of several drugs currently used in clinical practice for their potentiation of the antifungal effect of the fungistatic agent fluconazole (FLC) on *Candida albicans*. This facilitated the discovery of several compounds, such as inhibitors of the calcineurin [247, 248] or Tor pathways [249–251], efflux pump inhibitors (derived compounds of milbemycin) [252–254], and more recently, antibodies against heat-shock 90 protein (HSP90) [255]. In particular, inhibitors of the calcineurin pathway were shown to be fully active *in vivo* in the potentiation of fluconazole, and they also led to a dramatic decrease in fungi virulence [256–260].

Systematic screening of chemical compounds libraries was also undertaken, essentially by industrial laboratories as an attempt to discover new antifungal compounds. High throughput screening of the legacy Schering-Plough compound collection has recently lead to the discovery of a new glucan synthase inhibitor effective again *C. albicans* and *C. glabrata* [261–263].

Some analysis used reverse genetic assay in which, *C. albicans* heterozygous deletion or transposon disruption mutants collection were screened for growth under treatment with collections of chemical compounds [264, 265]. This approach allowed identification of both antifungal drugs and the genes related to the mechanism of action of the related compounds.

Another type of high-throughput screens of chemical libraries was achieved measuring the viability of drug-treated* Caenorhabditis elegans* infected with *C. albicans* [266]. Compounds can be simultaneously screened for antifungal efficacy and host toxicity, which overcomes one of the main obstacles in current antimicrobial discovery. A pilot screen for antifungal compounds using this novel *C. elegans* system identified 15 compounds that prolonged survival of nematodes infected with the medically important human pathogen *C. albicans*. One of these compounds, caffeic acid phenethyl ester (CAPE), had effective antifungal activity in a murine model of systemic candidiasis and had *in vitro* activity against several other fungal species [266]. In addition, this whole-animal system may enable the identification of compounds that modulate immune responses and/or affect fungal virulence factors that are only expressed during infection.

### 4.2. Genome-Wide Studies to Detect Potential New Antifungal Targets

The improvement of already existing antifungal drugs and the limitation of drugs resistance apparition has helped to elucidate the basic biology of the fungal pathogen. For this purpose, several groups made efforts to develop collection of systematic mutants essentially for *C. albicans*. An important difficulty for antifungal therapy is to develop drugs that exploit factors unique to fungi, which can be challenging considering that organism are eukaryotic and share many conserved biological pathways. Genes that are essential to fungal survival are possible targets for drug development.

Using the GRACE (gene replacement and conditional expression) or CPR (conditional promoter replacement) technologies, some research groups have assessed the essentiality of *C. albicans* and *Aspergillus fumigatus* genes [267, 268]. One study identified 567 essential genes in *C. albicans* [267]. And another study screened 54 genes of *A. fumigatus* based on ortholog functions and essentiality in *C. albicans* and *S. cerevisiae* [268], of which 35 were defined as essential in *A. fumigatus*. Authors were able to show that while the ERG11 gene family (CYP51A and ERG11B) is essential in *A. fumigatus*, the individual genes themselves are not. These analyses provide interesting and fully informative data for antifungal drug design and improve upon previous *in silico* analyses that when using *S. cerevisiae* data were only able to identify 61% of homologous genes reported in the genes found in the Roemer et al. analysis [267].

The diploid state of the genome presents a major problem to the development of a mutant collection. Therefore, some collections consist of heterozygous deletion [265] or transposon disruption mutants [264, 265]. Other collections contain homozygous transposon disruption mutants based on the random insertion thanks to the Tn7 transposon to a UAU cassette [269, 270]. These collection were first restricted to the transcription factors of *C. albicans* [269, 271, 272] but continue to be enlarged for the entire genome [270, 273]. Other collections consist of deletion mutants constructed with PCR generated deletion cassettes, with two different markers for each allele in the case of *C. albicans* [274, 275]. Such collections are now being constructed for *C. glabrata*.

Three kinds of analyses detailed below were performed with these collections. They aimed a better understanding of the modifications occurring in the fungi submitted to antifungal treatments or of the relationship developed between the fungus and its host all along the infectious process. Such knowledge might improve the actual therapy to avoid resistance development or might allow playing on the host-fungus equilibrium to improve recovery of patients.

First of all, treating strains with already known antifungal drugs and analyzing for example, growth modification and later transcriptional rewiring, some authors try to better understand drugs mechanisms of action and/or to find synergistic effect between them [272, 274]. Gene encoding the transcription factor Cas5 was found to be involved in the response to caspofungin [272]. Other studies showed that *AGE3*, which encodes an ADP-ribosylation factor GTPase activating effector protein, if deleted, abrogates fluconazole tolerance in *C. albicans*. Interestingly, Brefeldin A, an inhibitor of ADP-ribosylation factor, resulted in a synergistic effect with other drugs for *C. albicans* as well as for *Aspergillus* [273]. Finally, Homann et al. screened a collection of 143 transcription factor mutants under 55 distinct conditions among which exposition to fluconazole and 5FC, and they conclude in their analysis that nearly a quarter of the knockout strains affected sensitivity to commonly used antifungal drugs [274].

Other studies were geared better understand the biology of fungal species. For this purpose, mutant collections were subjected to a wide range of environmental conditions, modifying elements such as pH, salt concentration, carbon sources, oxidative conditions, temperature, and availability of essential elements such as metals (iron, copper, zinc, etc.) [274, 276].

Understanding the relationship between fungus and host during infection may provide further information useful for the improvement of antifungal treatment. In order to analyze the cross-talk occurring between fungus and host during the infectious process, researchers screened the colonization properties of mutants directly in hosts. One study that was performed with 1201 gene knockout mutants of *Cryptococcus neoformans* analyzed their *in vivo* proliferation profile in the murine lung, and they were able to identify 40 infectivity mutants [277]. Gene deletions in these mutants were previously uncharacterized and did not show any defect in traits known to be linked to virulence (polysaccharide capsule formation, melanization, and growth at body temperature). At least, four other similar studies were performed with *C. albicans* mutants. Two of them were done in invertebrate host models such as *C. elegans* or *D. melanogaster *[266, 278]. Interestingly, the *Cas5* Δ/Δ mutant, which has already been shown to be important for caspofungin response, was shown to be less virulent in both invertebrate models of infection [278, 279]. Finally, this transcription factor was demonstrated as crucial for cell wall integrity, and its importance in virulence was confirmed in the mice intravenous model of infection [278]. Two other studies screened collections of *C. albicans* mutants directly in mice by pools of mutants that were previously tagged [280, 281]. One collection was restricted to Zn2-Cys6 transcription factors (TF) mutants and the other was composed of mutants affecting about 11% of the entire *C. albicans* genome with no respect to a gene class. In both cases, mutants were also screened for traits known to be linked to virulence, such as the ability to filament and proliferate as well as the ability to grow at 42°C, at high and low pH, and in oxidative conditions. Noble et al. identified 115 mutants among the 674 screened with attenuated infectivity, but normal morphological switching and proliferation [281]. More precisely, they identified glycolipid and glucosylceramide as the first small molecules synthesized by *C. albicans* that are specifically required for virulence. Vandeputte et al. identified two Zn2-Cys6 TF mutants within 77 tested. These mutants displayed attenuated infectivity in their pool test, which was also confirmed in independent single strains infection of mice *ZCF13* and *ZCF18* [280]. Both genes were previously uncharacterized. *ZCF18* showed a slight growth defect in contrast to *ZCF13* which grew normally at body temperature, but slightly less at 42°C. *ZCF13* mutant displayed an abnormal morphology, producing strongly filamentous colonies on YPD medium at 35°C and displaying high invasion ability. *ZCF18* deletion also led to a slight enhancement of colony wrinkling. Both genes are currently under further analysis.

Unfortunately, whenever promising, up to now, no new compound and/or new target have been selected for further development from these approaches.

## 5. Conclusion

These last years were very rich in better knowledge of molecular basis of antifungal resistance and more generally of the metabolism of pathogenic fungi. Antifungal drug resistance appears to essentially be due to point mutations in either drug targets or transcription factors regulating actors of the resistance. In the near future, high throughput diagnostic tools could be used in the course of treatment of fungal infections in order to detect resistance and adjust therapeutic strategies accordingly before any clinical evidence and therefore allow a rapid adjustment of the antifungal treatment.

One of the challenges of finding new antifungal targets in *C. albicans* was the lack of sophisticated screening technologies often employed with other fungal species such as *Saccharomyces cerevisiae*. The recent application of genome-wide studies to pathogenic fungi for both host-pathogen interactions and the biological study will hopefully encourage and facilitate the development of new effective therapeutic strategies. Such improvements in antifungal treatment may lead to a better clinical outcome.

## Figures and Tables

**Table 1:** Characteristics of main fungal infections worldwide.

Body location	Pathogen type	Organ	Most frequent genus	Estimated incidence of infection*
superficial	primary	Skin and hair	*Malassezia*	~140,000,000 cases/year
cutaneous	primary	Skin and nails	Trichophyton *Epidermophyton * * Microsporum *	~1,500,000,000 cases/year
mucosal	opportunistic	Vagina, digestive tract, urinary tract and	*Candida*	~75,000,000 cases/year ~9,500,000 cases/year
		eye	* Aspergillus, Fusarium*	~1,000,000 cases/year
systemic	opportunistic	any organ (lungs, brain, bloodstream etc.)	*Candida * *Aspergillus * *Cryptococcus * * Histoplasma * *Pneumocystis * * Coccidioidomyces * and so on	~300,000 cases/year ~350,000 cases/year ~1,000,000 cases/year ~500,000 cases/year >200,000 cases/year up to 300,000 cases/year

*adapted from “The Fungal Research Trust. How common are fungal diseases? Fungal Research Trust 20th Anniversary meeting. London June 18th 2011.”

**Table 2:** Nature, target, mode of action, and fungal resistance mechanisms of the major antifungal drugs used in human therapy.

Antifungal agent	Mode of action and cellular target	Mechanism of resistance
polyenes	binding to ergosterol	absence of ergosterol (loss of function mutation in *ERG3* or* ERG6*)
		decrease of ergosterol content in cells

azoles	inhibition of cytochrome p450 function: 14*α*-lanosterol demethylase (*ERG11*) sterol Δ^22^ desaturase (*ERG5*)	efflux mediated by multidrug transporters
		decrease of affinity in Erg11p by mutations
		upregulation of *ERG11 *
		alterations in the ergosterol biosynthetic pathway

allylamines	inhibition of squalene epoxidase (*ERG1*)	unknown

morpholines	inhibition of sterol Δ^14^ reductase (*ERG24*) and the Δ^7–8^ isomerase (*ERG2*)	unknown

5-fluorocytosine	inhibition of nucleic acids synthesis	defect in cytosine permease
		deficiency or lack of enzymes implicated in the metabolism of 5-FC
		deregulation of the pyrimidine biosynthetic pathway

echinocandins	inhibition of *β*-1,3 glucan synthase (*FKS1&2*)	alteration of affinity of echinocandins for *β*(1,3)-glucan synthase

**Table 3:** *Cis*-acting elements involved in drug resistance.

	Organism	Gene	Regulatory element		Position respectively to the ATG	*Trans-*acting factor
			Name	Sequence		
ABC transporters			BEE	—	−960 to −710	?
			DRE	ACGGATATCGGATATTTTTTT	−460 to −439	Tac1
		*CDR1*	NRE	CTGATTGA	−335 to −328	?
	*C. albicans*		SRE1	GGAGTAGCAAGTGTGTCAAGAACCTGAATTC	−740 to −711	?
			SRE2	TTATCCGAAACGCTTTACTCCTCTATTATT	−691 to −661	?
		*CDR2*	DRE	ACGGAAATCGGATATTTTTTT	−221 to −201	Tac1
	*C. glabrata*	*CgCDR1*	PDRE	TTCCGTGGAA	−1201 to −1192	CgPdr1
		*CgCDR2*	PDRE	TTCCGTGGAA	−560 to −551	CgPdr1

MFS transporters			HRE/YRE	—	−561 to −520	Cap1/?
			BRE/ MDRE	ACGGTAAAATCCTAATTGGGAAAAATACCGAGAATGA	−296 to −260	Mcm1/Mrr1
	*C. albicans*	*CaMDR1*	AR1	—	−397 to –301	?
			AR2	—	−588 to –500	?
			AR3	—	−287 to −209	?
	*C. glabrata*	*CgFLR1*	YRE3	TTAGTAA	−372 to −366	CgAp1

ERG11	*C. albicans*	*ERG11*	ARE	AATATCGTACCCGATTATGTCGTATATT	−224 to −251	Upc2
	*C. glabrata*	*ERG11*	SRE			Upc2A
